# Characterization of the complete mitogenome of *Gymnocypris dobula* (Günther, 1868) (Cypriniformes: Cyprinidae)

**DOI:** 10.1080/23802359.2021.1972051

**Published:** 2022-01-30

**Authors:** Jiasheng Li, Shiyi Chen, Tashi Lahm, Ying Peng, Haodi Shen, Kun Zhang, Wenhua Huang, Xudong Liang, Bingjian Liu, Meiqun Chen

**Affiliations:** a National Engineering Research Center for Marine Aquaculture, Zhejiang Ocean University, Zhoushan, China; b Institute of Fisheries Science, Tibet Academy of Agricultural and Animal Husbandry Sciences, Lhasa, P. R. China

**Keywords:** *Gymnocypris dobula*, mitochondrial genome, phylogenetic relationships

## Abstract

*Gymnocypris dobula*, classified into the highly specialized Schizothoracine fish, is endemic to Tibet, China. The complete mitochondrial DNA sequence of *G. dobula* was 16,728 base pairs in length and comprised 22 transfer RNA genes, 13 protein-coding genes, two ribosomal RNA genes as well as one control region as in a typical vertebrate mitochondrial DNA gene. The ML and BI trees showed that *G. dobula* was most closely related to *Gymnocypris scleracanthus* within the highly specialized group. This mitogenome provides new molecular data for further taxonomic and phylogenetic studies of Schizothoracine fish.

The Schizothoracine fish, one of three broad fish lineages (including the Glyptosternoids and *Triplophysa*) generally discovered on the Qinghai-Tibetan Plateau, plays an essential ecological role in the plateau ecosystem (Ma et al. 2015). *Gymnocypris dobula* (Cypriniformes: Cyprinidae) is endemic to Tibet, China, and has a high economic value to marginal fishermen (Chan et al. 2016). The unique climatic and geographical characteristics of the Qinghai-Tibet Plateau resulted in the complex phylogenetic relationship among the schizothoracinae fish (Liang et al. 2017; Quan et al. 2021). Based on its morphological characters without any scales and barbels, *G. dobula* was classified into the highly specialized group of Schizothoracine fish (Qi et al. 2012). Owing to its distribution only in several localities, *G. dobula* is assessed as Vulnerable (VU) status by International Union for Conservation of Nature (IUCN). In this study, the complete mitochondrial DNA sequence of *G. dobula* was reported contributing to a better understanding of its further genetic studies, and also providing significant information for the reference of systematics and conservation.

The *G. dobula* sample was collected from Yanghu Lake (N 29.00°, E 90.41°), Tibet, Chian, and was stored in National Engineering Research Center for Marine Aquaculture, Zhejiang Ocean University (Jian, Chen and 1522490198@qq.com) under the voucher number F20200113. Genomic DNA was extracted from muscle using the standard phenol/chloroform extraction method (Sambrook and Russell 2006). The mitochondrial sequences, amplified by PCR with seventeen pairs of primer (Table S1), were obtained through Sanger dideoxy sequencing and assembled by CodonCode Aligner 5.1.5. The assembled mitochondrial genome was annotated using the online tool MITOS (http://mitos2.bioinf.uni-leipzig.de/index.py) (Bernt et al. 2013) and software Sequin (version 13.70, http://www.ncbi.nlm.nih.gov/Sequin). The annotated sequence was deposited in GenBank with the accession number MW924117.

Similar to the typical mitogenome of vertebrates, the mitogenome of *G. dobula* was a closed double-stranded circular molecule of 16,728 bp, including 13 protein-coding genes (PCGs), 22 tRNA genes, two ribosomal RNA genes (12S and 16S rRNA), and a control region (Boore 1999; Zhang et al. 2017). Most mitochondrial genes, including 12 PCGs, 14 tRNAs, and two rRNAs were encoded on the H-strand except for ND6 and eight tRNA genes (Gln, Ala, Asn, Cys, Tyr, Ser^UCA^, Glu, and Pro), which are encoded on the L-strand. The overall base composition was A (28.46%), T (27.21%), C (26.06%), G (18.27%), respectively, which showed a negative GC-skew value (–0.176) and positive AT-skew value (+0.023). The nucleotide composition of the whole mitogenome was A + T biased (55.67%), and the A + T content of PCGs, tRNAs, and rRNAs was 56.12%, 54.55%, and 53.60%, respectively. As with other vertebrate mitogenomes, most of these PCGs started by ATG initiation codon, except for COI by GTG (Zhang et al. 2020a). As for the stop codon, seven PCGs performed the routine termination codon (TAA or TAG), whereas five other PCGs (ND2, COII, ND3, ND4, and Cyt b) stoped with an incomplete stop codon T and one PCG (COIII) stoped with an incomplete stop codon TA. All of the tRNAs were predicted to be folded into canonical cloverleaf secondary structures except for tRNA-Ser^AGC^ using the online tool tRNAscan-SE (Lowe and Chan 2016). The 12S rRNA and 16 rRNA genes were 960 bp and 1,682 bp, respectively, which were typically separated by tRNA-Val. The length of the control region was 938 bp, with highly A + T (63.11%) rich.

Based on the sequences of 13 PCGs including 20 species of Schizothoracine fish and two outgroup species, we constructed maximum likelihood (ML) and Bayesian inference (BI) phylogenetic trees. The ML and BI trees were constructed by the software PhyML 3.0 (Guindon et al. 2010) and MrBayes 3.2.6 (Ronquist et al. 2012), respectively, with GTR + F + I + G4 as the best-fit evolutionary model determined by ModelFinder (Kalyaanamoorthy et al. 2017). The two trees showed the identical topology structure and indicated that *G. dobula* was closely related to *Gymnocypris scleracanthus* (Figure 1). The genus *Gymnocypris* was polyphyletic and clustered into the highly specialized group, which was consistent with previous studies (An et al. 2020; Zhang et al. 2020b).

**Figure 1.**
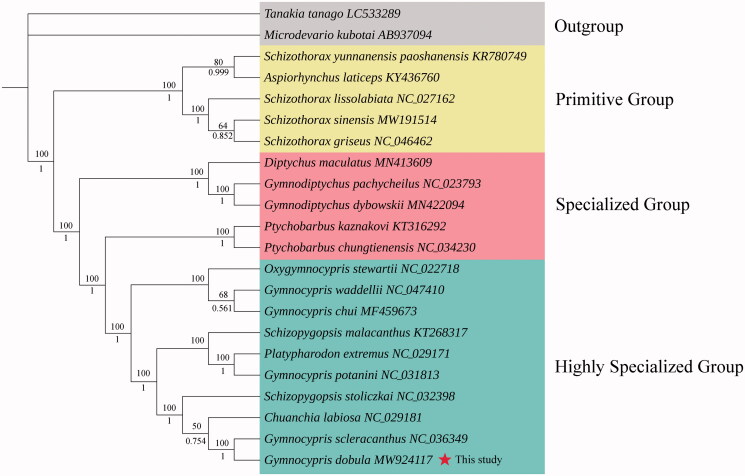
Phylogenetic analysis based on the sequences of the 13 PCGs in the mitogenome. ML tree with bootstrap values (above, with 100,000 replications) and BI posterior probabilities (below, with 100,000 generations) were shown next to nodes. The number after the species name was the GenBank accession number. The genome sequence in this study is labeled with a red star.

## Supplementary Material

Supplemental Material

## Data Availability

The genome sequence data that support the findings of this study are openly available in GenBank of NCBI at (https://www.ncbi.nlm.nih.gov/nuccore/ MW924117) under the accession number: MW924117.
