# Supplementary material for: Characterization of the complete mitogenome of Gymnocypris dobula (Günther, 1868) (Cypriniformes: Cyprinidae)
Source: Mitochondrial DNA B Resour. 2022 Jan 30;7(1):297–9. doi: 10.1080/23802359.2021.1972051 (PMC8812766; doi:10.1080/23802359.2021.1972051)
Supplement: Supplemental Material [file TMDN_A_1972051_SM0284.docx]

**Supplementary table**

Table S1 Primers used for the amplification and sequencing of the mitogenome of *G. dobula*.

| Fragment No. | Primer name | Sequence (5’-3’) |
| --- | --- | --- |
| F1 | GDF1 | GACTTCTACGATTCTACTCTG |
|  | GDR1 | GTGATTGAGCTAGCTGTTTG |
| F2 | GDF2 | CAGCATTGTACCATTCACATG |
|  | GDR2 | GAGACAAGTGATTGCGCTAC |
| F3 | GDF3 | CTTGAGCCGTTTGTGTTCGGAG |
|  | GDR3 | GCAAGGGTGAAGGCAAGCATG |
| F4 | GDF4 | GTCACCGGAAGGATTGTAATC |
|  | GDR4 | GTGCCTAGTCCCAGACTAGAG |
| F5 | GDF5 | CTTGGGTATGAGTTCTCTAG |
|  | GDR5 | GGTCTGATGTTATCCTAAG |
| F6 | GDF6 | GATACAATGCGTTATTGAGAATG |
|  | GDR6 | GTAATTCCGGCAGCTAAGACAG |
| F7 | GDF7 | GTGATGTAGATCGTCCACAGAG |
|  | GDR7 | CTAATGTCTTCATAAGGAAGTG |
| F8 | GDF8 | GCTCTTCACGACAGGCATC |
|  | GDR8 | CATGGTCAGTTTCAGGATTC |
| F9 | GDF9 | GCTGGTGGAGATCACTTTACG |
|  | GDR9 | GTGTTGGTGCTAAGCTTGCATG |
| F10 | GDF10 | GATGAAGAGTGGTATAAGGTC |
|  | GDR10 | GCTAGACCTATTAGGCCTAG |
| F11 | GDF11 | GTTGTCGTCAGGATTAGAATG |
|  | GDR11 | GAACTGCCGCTAGTACTATTG |
| F12 | GDF12 | GATATGTTGAGCTGAGGAC |
|  | GDR12 | GTCCAATGTAGTAAGTAGG |
| F13 | GDF13 | CAAGTAGGTAACCAGAATC |
|  | GDR13 | GATGGTGAAGTAGGTCGAG |
| F14 | GDF14 | GTTGTAGTTCAGTCGATCCG |
|  | GDR14 | CTTACTCGGCAGCCTTGGCTG |
| F15 | GDF15 | GTCGATGTGGTGATTATAG |
|  | GDR15 | GTCTGAGTTGAGCCCAATC |
| F16 | GDF16 | CAAGAGTCATCTATTACGCTG |
|  | GDR16 | GATTCCATCATGCATGATGTG |
| F17 | GDF17 | GATTATGTACATACATAATAGTG |
|  | GDR17 | GCGGAGACTTGCATGTGTAAG |
